# Enrichment of Biscuits with Matcha Green Tea Powder: Its Impact on Consumer Acceptability and Acute Metabolic Response

**DOI:** 10.3390/foods7020017

**Published:** 2018-02-01

**Authors:** Benjapor Phongnarisorn, Caroline Orfila, Melvin Holmes, Lisa J. Marshall

**Affiliations:** 1 School of Food Science and Nutrition, University of Leeds, Leeds LS2 9JT, UK; nbjp459@hotmail.com (B.P.); prcmjh@leeds.ac.uk (M.H.); 2 Faculty of Agricultural Technology, Phuket Rajabhat University, Phuket 83000, Thailand

**Keywords:** Matcha green tea powder, catechin stability, consumer acceptability, acute metabolic response, functional food

## Abstract

Matcha green tea powder (MGTP) is made with finely ground green tea leaves that are rich in phytochemicals, most particularly catechins. Shortbread biscuits were enriched with MGTP and evaluated for consumer acceptability and potential functional health properties. Baking decreased the content of total catechins by 19% compared to dough, although epimerization increased the amount of (+)-gallocatechin gallate at the expense of other catechins such as (−)-epigallocatechin gallate. Consumer acceptability tests using a 9-point hedonic scale showed that consumers preferred enriched biscuits with low content of MGTP (2 g of MGTP 100 g^−1^ of flour), and an increase of sugar content did not significantly improve the acceptability of MGTP-enriched biscuits. Overall, enrichment of biscuits with MGTP did not significantly affect the postprandial glucose or triglyceride response (area under curve) compared to non-enriched biscuits consumed with water or MGTP drink. Enriching biscuits with Matcha green tea is acceptable to consumers, but may not bring significant postprandial effects.

## 1. Introduction

**Figure 1:**
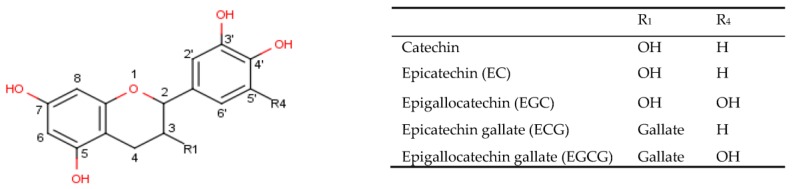
Structure of the main catechins found in green tea.

Catechins are the main polyphenols found in green tea. As shown in Figure 1, catechins have a common carbon backbone with variations in the substitutes at the C-3 and C-5′ positions [1]. The four major catechins found in green tea are (−)-epigallocatechin (EGC), (−)-epicatechin (EC), (−)-epigallocatechin gallate (EGCG), and (−)-epicatechin gallate (ECG). Their trans epimer forms, namely (+)-catechin, (+)-gallocatechin gallate (GCG), (+)-gallocatechin (GC), and (+)-catechin gallate (CG), are found in minor amounts. The epimerization from cis to trans is reversible and can occur when cis catechins are exposed to high temperatures [2]. EGCG is the most abundant catechin in green tea leaves and contributes to 50% of total catechins in tea leaves. Hence, it is been used as a quality indicator in green tea products [3]. It is also found at low levels in a range of foods including apples, red berries, nuts, and legumes.

Consumer research has suggested that even though green tea is unfamiliar to most non-Asian consumers, their desire to drink green tea is enhanced due to its perceived health benefits [4,5]. Moreover, there are also the demands for convenience products (i.e., biscuits) with value-added components [6,7]. Incorporating green tea powder into bakery products may mask the bitterness or astringency of green tea perceived by consumers. A few studies have incorporated green tea into bakery products and have shown effects on the physicochemical, colour, textural, and shelf life properties [8,9,10]. Studies found good sensory acceptability of cakes enriched with up to 20% green tea substituting wheat flour [9]. To date, there have been no reports on the potential physiological effects of green tea-enriched food products.

The catechins found in green tea have been shown to have potential roles in preventing cancer development, diabetes, and cardiovascular diseases [11,12]. Zhong et al. [13] found a 25% decrease in carbohydrate absorption after a consumption of green tea extract containing 300 mg of EGCG and 100 mg of ECG with a carbohydrate meal, suggesting that green tea may reduce the amount of glucose absorbed into the bloodstream, which in turn may lower the risk of developing diabetes [12,13]. Moreover, a review by Koo and Noh [14] suggested that green tea reduces the absorption of dietary fat by interfering with the processes of lipid digestion in the intestine. Studies using animal models have shown that green tea compounds can slow down lipid absorption by inhibiting the activity of pancreatic lipase [15,16]. Unno et al. [16] showed that 224 mg of green tea catechins consumed as tea infusion after meal consumption can lower the concentration of lipids in the blood by 15% and therefore may lower the risk of developing cardiovascular diseases. Studies so far have used green tea in the form of pure extracts or tea infusions to study the potential health effects, but none have used it as part of a functional food.

The aims of this study were to (1) investigate the effect of baking on the stability of catechins; (2) evaluate the sensorial acceptability of shortbread biscuits enriched with Matcha green tea powder (MGTP) and assess the effect of sugar addition on acceptability; and (3) investigate the effect of MGTP on the postprandial glucose and triglyceride response in human volunteers. We discuss the potential of MGTP-enriched biscuits as functional foods.

## 2. Materials and Methods

### 2.1. Materials

Matcha factory^®^ Matcha green tea powder (13.2% of total phenolic content, 10% total catechin) was purchased from Chah Ltd. (Solihull, UK). Tesco^®^ plain flour, Tate & Lyle^®^ caster sugar, and Anchor^®^ unsalted butter were purchased from Tesco supermarket (Leeds, UK).

HPLC (High Performance Liquid Chromatography)-grade acetonitrile and ethanol were purchased from Fisher Scientific Co. Ltd. (Leicestershire, UK). HPLC-grade methanol, (−)-epicatechin (EC), and caffeine were purchased from Sigma Aldrich Co. Ltd. (St. Louis, MI, USA). (−)-Epicatechin gallate (ECG), (−)-epigallocatechin (EGC), and (−)-epigallocatechin gallate (EGCG) were obtained from Cambridge Bioscience Ltd. (Cambridge, UK). (+)-Gallocatechin gallate (GCG) was purchased from Insight Biotechnology Co. Ltd. (Middlesex, UK). All water refers to deionized Millipore water, Millipore Ltd. (Hertfordshire, UK).

### 2.2. Preparation of Shortbread Incorporated with MGTP

The dough was prepared by beating butter (83.3 g) and sugar (at three different levels, 25, 30, or 35 g 100 g^−1^ of flour) together with a mixer (KM336 Chef Classic, Kenwood Ltd., Havant, UK) for 5 min at speed 4 (180 rpm), and then flour (100 g) and MGTP were added (at the level of 2, 4, or 6 g 100 g^−1^ of flour), followed by mixing for 5 min at minimum speed (50 rpm). The dough was wrapped in clingfilm and rested for 1 h at 4 °C. The dough was rolled to 0.4 mm thickness, and then the biscuits were cut into a circle shape (4.8 cm diameter) and rested for 10 min before being placed in a conventional oven (BC190.2TCSS, Baumatic Ltd., Merthyr Tydfil, UK) at 180 °C for 15 min. After baking, the biscuits were left to cool down at room temperature.

The weight of biscuits was measured before and after baking to calculate weight loss during baking. Samples of each biscuit were prepared for sensory evaluation. For HPLC analysis, the dough and shortbread biscuit samples (made with 25 g sugar at the three levels of 2, 4, or 6 g MGTP 100 g^−1^ of flour) were ground, kept overnight in a freezer (−20 °C), and then freeze-dried (Christ Alpha 1-4 LD, SciQuip Ltd., Shropshire, UK) and packed into vacuum-sealed bags until HPLC analysis.

### 2.3. Defatting of Samples and Extraction of Catechins

Freeze-dried sample was weighed (1 g), mixed with 30 mL of hexane, and placed in a heat circulating water bath (Grant Instruments, Cambridge, UK) at 70 °C for 20 min. After cooling down, samples were centrifuged at 3000 rpm for 10 min. The hexane fraction was decanted. The samples were left in the fume cupboard with the light off for 2–3 h to evaporate the hexane from the samples. Defatted samples were extracted by adding 25 mL of 70% methanol with 0.3% formic acid and incubated in a water bath at 70 °C for 5 min to equilibrate to the temperature and another 45 min to extract. After cooling down, the tubes were centrifuged at 3000 rpm for 10 min and the supernatant was collected. The extraction was repeated, the supernatant was pooled together, and its volume was made to 50 mL. The samples were filtered through a 0.2 µm PTFE (Polytetrafluoroethylene) syringe filter and put in amber vials for further HPLC analysis.

### 2.4. HPLC Analysis of Catechins in Dough and Biscuit Samples 

A method for HPLC analysis was adapted from Wang and Zhou [17]. The separation was performed on a Phenomenex C18 reverse-phase column (5 µm, 250 × 4.6 mm) (Phenomenex, Cheshire, UK) with a conventional HPLC coupled with a UV-Vis detector (SPD-20A, Shimadzu Corporation, Kyoto, Japan). A binary gradient of water with 0.1% formic acid (eluent A) and acetonitrile with 0.1% formic acid (eluent B) was used to run on the system at a flow rate of 0.5 mL·min^−1^. The gradient started at 10% B, remained at 10% for 5 min, and increased to 15% over 9 min, then climbed to 60% over 23 min, then to 95% over 3 min, and remained at 95% for 2 min to wash the column before returning to 10% over 2 min and re-equilibrating over 6 min. The total length of the method was 45 min. The column temperature was set at 25 °C. Catechins and caffeine were detected at 275 nm. Identification of each catechin was performed by comparing the retention time and spectrum with the standard. Standard curves for quantification were prepared with standard compounds dissolved in extracting solvent (70% methanol with 0.3% formic acid) at concentrations between 2.5–100 μg·mL^−1^. The standards were caffeine, EC, EGCG, ECG, EGC and GCG.

### 2.5. Sensory and Acceptability Studies

Ethical approval for the sensory evaluation study was granted by the Mathematics, Physical Sciences, and Engineering Ethical Committee at the University of Leeds (MEEC 13-026). Sensory evaluation of biscuits was conducted by human panelists, who scored the biscuits using a 9-point hedonic scale. The attributes tested were overall acceptability, appearance, aroma, colour, texture, bitterness, and sweetness. All sensory evaluation sessions were carried out in separate booths equipped with a Compusense 5.6 sensory software (Compusense Inc., Guelph, ON, Canada), in which responses were recorded directly by the participants.

The tests evaluated 9 shortbread formulations with 3 levels of MGTP and 3 levels of sugar. Samples were assigned with 3-digit codes, and the software randomised their serving orders. Plain water was provided to rinse the mouth between samples. The evaluations were repeated in two sessions with same participants. Fifty-four participants completed the first session, and 46 participants completed the second session.

### 2.6. Human Intervention Study for Study of Metabolic Response

Ethical approval for this part of the study was granted by the Mathematics, Physical Sciences, and Engineering Ethical Committee at the University of Leeds (MEEC 14-040). The study was designed according to the FAO (Food and Agriculture Organization) protocol [18]. Healthy adults (*n* = 4 were Asian, *n* = 3 were South American, *n* = 2 were European, *n* = 1 was African) were recruited to the study after they met the eligibility criteria according to a health screening questionnaire (18–60 years; not allergic to any food; not pregnant or lactating; not diagnosed with chronic diseases such as diabetes, cancer, cardiovascular or digestive diseases; not taking any medication that affect the glucose and triglyceride response). The sample size was decided based on harmonized GI methodology [19] who established that 10 individuals is probably enough in most cases to obtain a significant difference between. The design of the study was a cross-over randomised control study without blinding with a one week washout period between sessions. Each participant was asked to attend following overnight fasting overnight for a least 10 h but was not asked to modify their usual diet.

#### 2.6.1. Reference and Test Meals

Three meals (one reference and two test meals) were tested by all panelists in this study in a random order. The reference meal consisted of 100 g of plain shortbread biscuits consumed with 300 mL of warm water. The two test meals consisted of either 100 g of MGTP-enriched biscuits consumed with 300 mL of warm water or 100 g of plain shortbread biscuits consumed with 200 mL of MGTP consumed as a tea drink followed by 100 mL of warm water (total liquid volume of 300 mL). The test meals contained 54–55g of available carbohydrate, of which 16.0 g was sugar and 36 g was fat. The MGTP-enriched biscuit contained 6 g of MGTP (catechin dose of 233 mg). Plain biscuits were prepared according to exactly the same procedure but without addition of MGTP. Once the biscuits were baked and cooled down, 100 g of biscuits were weighed and packed in vacuum-seal plastic bags and used within 1 month for the human study. Matcha green tea drink was made before serving by mixing MGTP (3 g, catechin dose of 257 mg) with 200 mL of warm water. All meals contained around 54–55 g of available carbohydrate. The composition information for the biscuits tested can be found as Supplementary Material (Table S1).

#### 2.6.2. Glucose and Triglyceride Measurement

Capillary blood glucose and triglyceride concentrations were measured from a finger-prick blood sample. A finger was sanitized with an antiseptic wipe before perforation of the skin with a Safe T pro Accu chek disposable lancet. The blood droplet was loaded onto a glucose test strip (Accu-Chek compact 17-drum test strips, Roche diagnostic Ltd., West Sussex, UK) and inserted into a glucometer (Accu-Chek^®^ Aviva blood glucose meter, Roche diagnostic Ltd., West Sussex, UK), which returned the blood glucose concentration in mmol·L^−1^. Another blood droplet was collected by a sterile disposable pipette (15 µL Safetec Pipettes, BHR Pharmaceutical Ltd., Nuneaton, UK). The extracted blood was transferred to a triglyceride test strip (CardioChek^®^ PTS Panel triglyceride test strips, BHR Pharmaceutical Ltd., Nuneaton, UK), which was inserted into a Cardiochek Professional analyser (CardioChek^®^ PA Blood Analyser, BHR Pharmaceutical Ltd., Nuneaton, UK), to measure the blood triglyceride concentration in mmol·L^−1^.

Overnight fasted blood glucose and blood triglycerides were measured at baseline; then, the subject was provided the meal to be consumed within a period of 10 min. Eight blood glucose measurements were taken at 15, 30, 45, 60, 90, 120, 150 and 180 min after the start of the food consumption. Three blood triglyceride measurements were taken at 60, 120 and 180 min after the start of the food consumption.

#### 2.6.3. iAUC Calculation

The incremental area under the curve (iAUC) of the glucose and triglyceride response was calculated according to FAO [18], using the trapezoidal rule in which all the areas of glucose response collected during the three hours period are added together, by ignoring the area beneath the baseline.

### 2.7. Data Analysis

Results were analysed statistically to determine mean values, standard deviations (STDs), and standard error of means (SEMs) of quantified masses of compounds obtained from HPLC in duplicate runs. The total catechins content was presented by the sum of the amounts of individual catechins (EGCG, ECG, EGC, GCG and EC). Mann-Whitney U Test was performed to determine the difference between before and after baking of catechin content.

For acceptability sensory results, one factor analysis of variance (ANOVA) with a significant level of *p* < 0.05 and Tukey’s-b post-hoc test was performed to determine the difference in scores between the different biscuit formulations. Two factor ANOVA with a significant level of *p* < 0.05 and Tukey’s-b post-hoc test was conducted to determine the difference of the acceptability between MGTP and sugar incorporated and their interaction effects. The response surface methodology was conducted using R program (R version 3.2.5, with R Commander package) to plot response surface and contour plot that explained the relationship between the independent variable: MGTP (X_1_) and sugar (X_2_); response variables; and testing acceptability attributes (Y): overall appearance, aroma, colour, texture, bitterness, and sweetness of the biscuit samples.

For the human metabolic study, one-way ANOVA and Tukey’s-b post hoc test was conducted to investigate the difference between test meals that affects the glucose response and triglyceride response.

Statistical analyses were carried out using the SPSS 22.0 statistical software package (SPSS Inc., Chicago, IL, USA).

## 3. Results and Discussion

### 3.1. Stability of Catechins and Caffeine During the Baking Process

**Figure 2:**
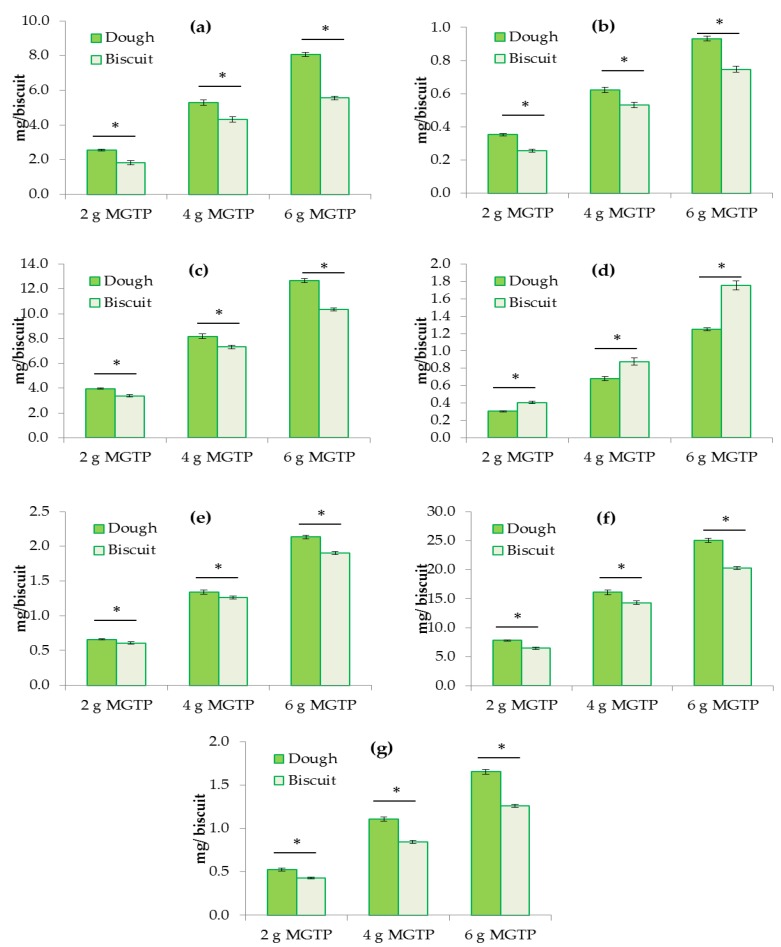
Amount of catechins in dough and shortbread enriched with 3 levels of MGTP (2, 4, 6 g 100 g^−1^ of flour): (**a**) EGC; (**b**) EC; (**c**) EGCG; (**d**) GCG; (**e**) ECG; (**f**) total catechins and (**g**) caffeine. Results are expressed as mg per biscuit. * indicates a significant difference (*p* < 0.05) before and after baking. The error bars represent the SEM (standard error of the mean) (*n* = 12 biscuits).

Biscuits with three different levels of MGTP were prepared (see Figure S1). Catechins and caffeine content in dough and biscuits were measured by HPLC-DAD (diode-array detector) (Figure 2, for chromatograms see Figure S2). The results indicate a significant loss of most catechins, except GCG, during the baking process, with a maximum loss of 19% in total catechins (Table 1). EGC presented the highest losses of 31% in biscuits enriched with the highest level of MGTP (6 g 100 g^−1^ of flour). However, there was an increase in GCG of up to 40% in the same biscuits, indicating that epimerization of EGC to GCG may have occurred during the baking process. Caffeine content also decreased during the baking process by up to 24%. The levels of total catechins in the highly enriched biscuits (6 g MGTP 100 g^−1^ of flour) were approximately 20–23 mg per one biscuit, which is around 9% of the amount present in a typical green tea infusion (257 mg 200 mL^−1^ cup).

The results are in agreement with the study by Wang and Zhou that showed an average retention of 83% for EGCG and 91% for ECG in green tea-enriched bread [17]. Epimerization of EGCG to GCG has been observed in bread [20]. Sharma and Zhou showed much higher losses of catechins during baking (up to 98%) [21]. The degradation followed first order kinetic parameters and could largely be prevented by acidification of the dough. Core temperatures of 120–130 °C achieved during baking would provide sufficient energy for epimerization of EGCG or EGC to GCG [21]. EGC is less stable than other catechins because of its radical scavenging hydroxyl group at the 5′ position of the B ring [22,23]. Therefore, this is could explain the increase in GCG in the MGTP-enriched biscuits.

### 3.2. Sensory and Overall Acceptability of Biscuits

**Figure 3:**
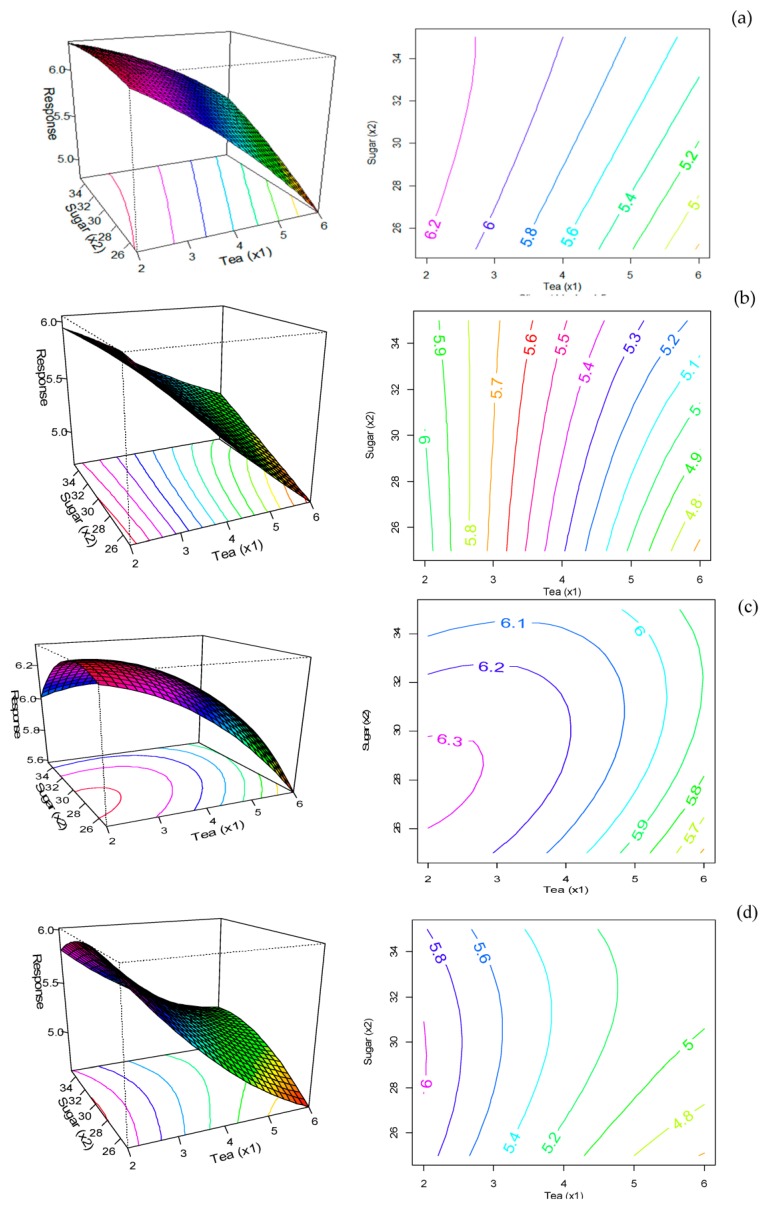
Response surfaces and contour plots of the effect of MGTP and sugar content incorporated (**a**) overall; (**b**) appearance; (**c**) aroma and (**d**) color acceptability of biscuits.

The response surface for overall acceptability (Figure 3a) shows that biscuits with low MGTP and low sugar content received the highest acceptability. The increase in sugar in biscuit formulation did not significantly affect the acceptability of the enriched biscuits. A similar response was observed for appearance (Figure 3b), with darker green and brown colour receiving lower acceptability scores. As shown in Figure 3c, the highest response of aroma acceptability was achieved around a sugar level of 30 g 100 g^−1^ of flour. The surface response method suggested the stationary point at 1.84 g of MGTP and 27.73 g of sugar 100 g^−1^ of flour to obtain the highest response. Heat treatment can cause undesirable aromas of green tea [24]. The trained panelist detected more undesirable (described as wet wood smell) aroma of heat processed green tea at 121 °C for 1 min compared to unprocessed green tea [24]. Therefore, the aroma of shortbread biscuits containing MGTP may be affected by baking temperature at 180 °C, as it will likely cause an undesirable aroma. However, Ahmad et al. [8] found that increasing green tea powder in cookies (1–4%) increased the aroma acceptability.

As shown in Figure 3d, the highest response of colour acceptability was for the biscuits with a sugar level of 30 g 100 g^−1^ of flour and low MGTP. The colour is thought to come from chlorophyll pigments, which can degrade at high temperatures. Kim et al. [25] suggested that green tea solution became less green and deeper yellow after heating at 120 °C for 4 min. Therefore, masking the colour of biscuits by adding fresh green colouring may increase the acceptability of biscuits.

**Figure 4:**
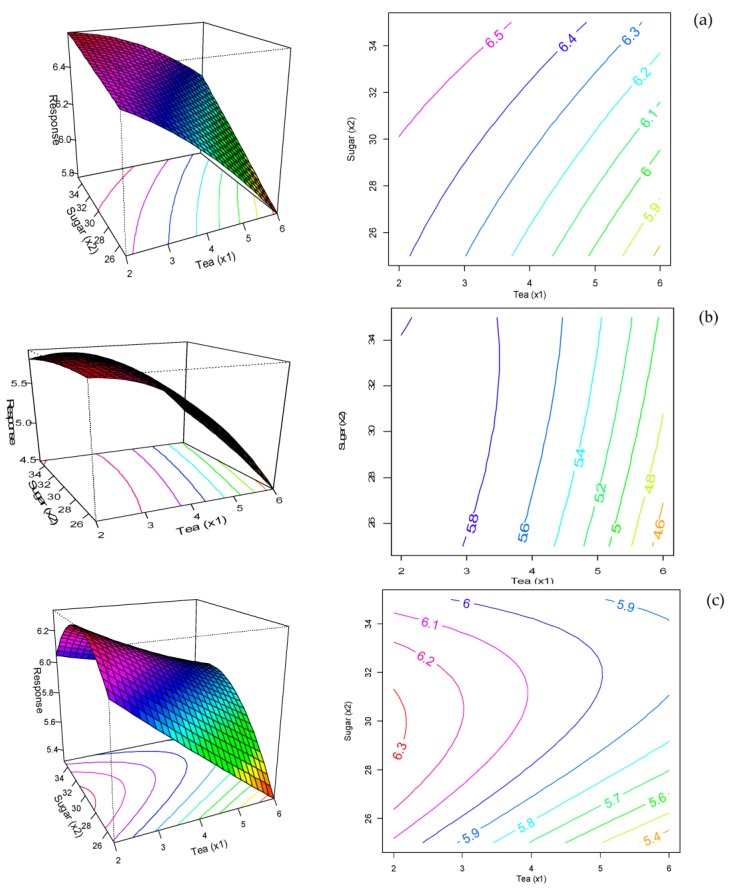
Response surfaces and contour plots of the effect of MGTP and sugar content incorporated (**a**) texture; (**b**) bitterness and (**c**) sweetness acceptability of biscuits.

Figure 4a shows that high sugar and low content of MGTP increased the texture acceptability with an optimum score for biscuits with 30 g sugar 100 g^−1^ of flour and low MGTP. Sugar increases the crunchiness of biscuits, while MGTP increases the hardness of biscuits (data is shown in supplementary doc, Figure S3). According to Figure 4b, the bitterness acceptability was significantly affected by the level of MGTP incorporated into the biscuit (*p* < 0.001). The bitterness acceptability received a high response with biscuits that had a low level of MGTP, and sugar did not improve the acceptability. Bitterness can significantly suppress the sense of sweetness and vice versa [26]. It was found that bitterness can be perceived at a much lower concentration than sweetness at a ratio of 1:31 magnitude [27]. Finally, as shown in Figure 4c, sweetness acceptability was significantly affected by the level of MGTP in the biscuit (*p* < 0.05), with higher acceptability for sugar content of 30 g 100 g^−1^ of flour for all MGTP levels tested. This indicates that sweetness does improve the acceptability of biscuits, particularly those with high MGTP. According to the sensory trial, appearance, color, and bitterness were the most important determinants of overall acceptability.

### 3.3. Metabolic Response to Matcha Green Tea Biscuits

In this pilot study, 10 subjects were recruited with ages ranging from 27 to 44 years and a mean BMI of 26.67 ± 4.48 kg·m^−2^, with a gender split of 6 females and 4 males. The portions of biscuits served to participants are shown in supplementary material (Figure S4).

#### 3.3.1. Glucose Response

**Figure 5:**
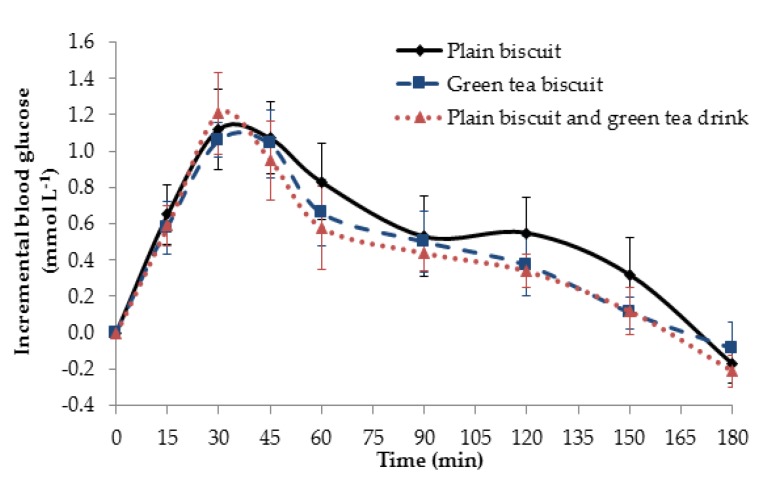
Mean incremental blood glucose response curves after ingestion of control and test meals over 180 min. (**-**◆**-**) represents the blood glucose curve of the control meal that consisted of plain biscuit consumed with warm water, (**-**■**-**) represents the blood glucose curve of the test meal with Matcha green tea biscuits consumed with warm water, and (**..**▲**..**) represents the blood glucose curve of the test meal with plain biscuits consumed with a Matcha green tea drink. Data expressed as the amount of blood glucose in mmol·L^−1^ and the error bars represent the SEM (*n* = 10).

Capillary blood glucose concentration was measured at baseline and every 15 min after consumption of the control or test meals. As shown in Figure 5, the glucose response of all of the food samples peaked at 30 min after meal intake, with a slow decrease until 180 min. Consumption of the control meal consisting of plain biscuits gave the highest average iAUC (106.1 ± 63.0 mmol·min L^−1^), followed by the test meal with MGTP-enriched biscuits (89.2 ± 44.7 mmol·min L^−1)^ and the test meal with plain biscuits and a MGTP drink (81.9 ± 36.0 mmol·min L^−1^). No significant differences in the iAUCs were observed between meals (one factor ANOVA). Although MGTP does have a tendency to lower iAUC, the variation between individuals is high. The response in the first 30 min appears to be the same amongst the foods, indicating that Matcha green tea does not affect rapidly available glucose (RAG), most probably from sucrose and rapidly digestible starch (RDS). The biggest differences appeared in later time points (>100 min), pointing to a potential inhibition of starch digestion by MGTP, whether in biscuit or tea form. There were significant differences in incremental blood glucose levels at time points 120 and 150 min post ingestion between the plain biscuits and the Matcha treatments.

Previous findings on the effect of green tea on glucose response have been inconclusive. Many in vivo and in vitro studies have suggested that green tea and EGCG acutely reduce postprandial blood glucose levels by inhibiting the activity of pancreatic α-amylase [28,29]. In an in vitro study, Forester et al. [28] found that EGCG non-competitively inhibited pancreatic amylase activity by 34%. Moreover, Tsuneki et al. [30] showed that glucose tolerance was significantly (*p* < 0.05) improved with tea administration compared with hot water during a glucose challenge in healthy humans. The authors speculated that the observed acute effects of green tea on blood glucose levels were mainly due to the promotion of insulin action in peripheral tissues, such as skeletal muscles and adipocytes via modification of serum protein [30]. Lochocka et al. [31] found that a single dose of green tea extract taken with a test meal significantly decreased starch digestion and absorption compared with the placebo treatment (*p* = 0.003). The study used a CO_2_ starch breath test, which measures the isotope ratio of _12_CO_2_/_13_CO_2_ in breath samples. The starch digestion and absorption was measured based on cumulative percentage _13_C dose recovery [31]. They found that green tea extract (257.6 mg of catechins) decreased starch digestion and absorption in humans when consumed with a test meal. The green tea extract was used in powder form (4 g of powder containing 257.6 mg of catechins) enclosed in a starch wafer, whereas the placebo was an empty starch wafer. In our study, we observed a significant difference between responses to plain biscuits and the two green tea treatments after 120 and 150 min, suggesting that catechins may have a mild effect on starch digestion. Josic et al. [32] found that green tea infusion (150.6 mg of catechins) showed no effects on glucose or insulin levels. An animal study suggested that EGCG acutely reduces postprandial blood glucose levels in mice when co-administered with corn starch, and this may be due in part to inhibition of α-amylase [28]. Moreover, a study on the effect of green tea supplementation on insulin sensitivity in rats suggested that regular green tea infusion drink could increase insulin sensitivity [33,34]. They also suggested that the amelioration of insulin resistance by green tea is associated with increased expression of glucose transporter IV (GLUT IV) found in adipose tissue. On the other hand, Park et al. [35] proposed that gallated catechins could elevate blood glucose levels by blocking glucose uptake into tissues. 

A factor that could have affected glucose response in the present study is the high fat content in shortbread biscuits. The fat content in food can lower the glucose response by slowing down gastric emptying [36]. Hence, the potential differences in starch digestion between control and test meals could have been masked by the high fat content in biscuits. In order to minimize the varied result from sugar and fat content in biscuits, the biscuits with less sugar and low fat content should be used to investigate the effect of MGTP on glucose response. We observed no effect of ethnicity on the glyceamic response to the biscuits. Previous studies have also not identified ethnicity as a significant factor to consider when testing glyceamic response [19].

#### 3.3.2. Triglyceride Response

**Figure 6:**
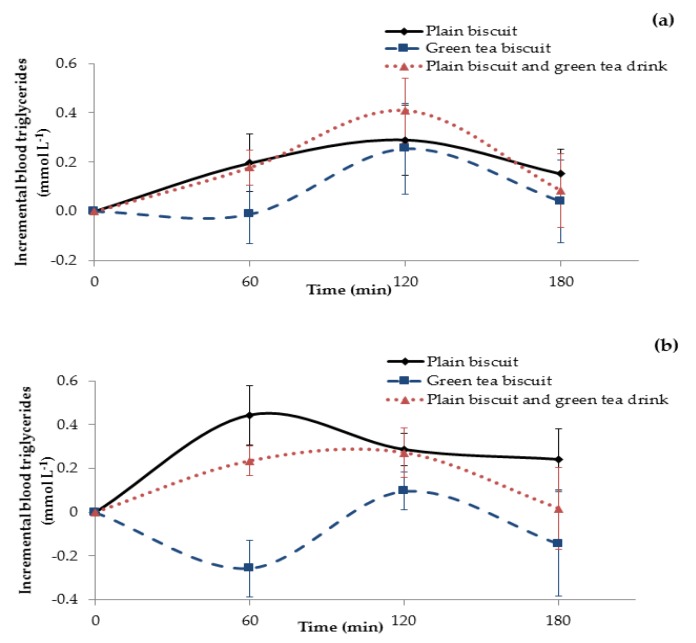
(**a**) The mean incremental blood triglyceride response curves after ingestion of control and test meals over 180 min (*n* = 10 participants); (**b**) the mean incremental blood triglyceride response curves after ingestion of control and test meals over 180 min among Asian subjects (*n* = 4 participants). (**-**◆**-**) represents the blood triglyceride curve of the control meal consisting of plain biscuits, (**-**■**-**) represents the blood triglyceride curve of the test meal with Matcha green tea biscuits, and (**..**▲**..**) represents the blood triglyceride curve of the test meal with plain biscuits consumed with a Matcha green tea drink. Data expressed as the amount of triglycerides in mmol L^−1^ and the error bars represent the SEM.

The incremental postprandial responses of plasma triglycerides after consumption of the control and test meals are shown in Figure 6a. When the participants consumed the control meal, plasma triglycerides rose from the baseline to reach the highest level at 120 min and almost returned back to baseline 180 min after consumption of the control meal. The concentration of triglycerides after the consumption of the test meal with Matcha green tea biscuits remained close to the baseline value until 60 min, then rose to a peak at 120 min and returned to baseline at 180 min. The reduction in iAUC after consumption of Matcha green tea biscuits (35.6 ± 44.6 mmol·min L^−1^) was observed, compared with the consumption of plain biscuits (44.6 ± 32.1 mmol·min L^−1^) and plain biscuits consumed with Matcha green tea drink (39.4 ± 35.7 mmol·min L^−1^). However, statistical analysis found that there was no significant difference in iAUC between all three food samples (*p* = 0.87) due to the high variation between individuals.

Figure 6b shows the incremental postprandial levels of plasma triglycerides after ingestion of the control and test meals for 4 Asian subjects compared to the other ethnicities. The curves show an average 0.26 mmol·L^−1^ decrease in plasma triglycerides following consumption of the test meal with the MGTP enriched biscuits, whereas the control meal with the plain biscuits induced an average 0.44 mmol·L^−1^ increase in plasma triglycerides. The iAUC of the test meal with Matcha green tea biscuits (8.0 ± 12.5 mmol·min L^−1^) was significantly lower (*p* = 0.02) than the other meals (51.7 ± 22.3 mmol·min L^−1^ and 32.7 ± 17.6 mmol·min L^−1^) for the control meal and test meal (plain biscuits with MGTP tea drink), respectively.

A previous study by Unno et al. [16] found that green tea drink consumed with a piece of bread covered with 20 g of butter lowered the postprandial triglyceride level compared to a meal with a control drink. In that study, there were 2 treatments of green tea; a moderate dose containing 224 mg of tea catechins and a high dose containing 674 mg of tea catechins. Both moderate and high dose treatments lowered the postprandial triglyceride levels and reduced the iAUC by 15.1% and 28.7%, respectively. Only the high dose showed a significant difference in iAUC compared to the control dose. Our findings are in agreement, as the dose of catechins (224 mg of tea catechins) reduced the iAUC by 15.1% but did not give a significant difference. The test meal used in the Unno et al. [16] study was lower in fat (18.8 g of fat) compared to our biscuits (35 g of fat).

The mechanism underlying the triglyceride lowering effect of green tea is mainly focused on the inhibition of the digestion of fat from the intestine [37]. Juhel et al. [38] found that green tea exhibits potential to inhibit gastric and pancreatic lipases. EGCG can enlarge fat droplets through interference with emulsification and micellar solubility of lipids [39,40,41]. Hence, the enlarged emulsion droplets can prevent the efficient emulsification of bile salt and reduce surface area for fat digestion by lipase [14,16,42,43]. Moreover, Suzuki et al. [37] proposed that catechins with gallate groups are located on the surface of the lipid emulsion and destabilize the lipid emulsion. The difference in responses according to ethnicity merits further investigation. Apolipoproteins are essential regulators of triglyeride circulation. Gene polymorphisms for the a1/c3/a4/a5 gene cluster were found to differ between Chinese and Caucasian populations [44]. Another study found that the postprandial triglyceride concentrations in South Asian men were significantly higher than European men [45]. This was indeed our observation in response to the plain biscuits with a significant effect of the green tea enrichment. These results need to be confirmed in a larger study stratified according to ethnic groups. Information about habitual diet, including catechin content, needs to be considered. Furthermore, more time points in the triglyceride time course need to be included, as triglyceride levels may take around 8 h to return to homeostatic levels.

## 4. Conclusions

This study showed that catechins in MGTP are relatively heat resistant, with only approximately 20% loss of total catechins during baking at 180 °C. Epimerization between EGC and GCG is thought to have occurred during biscuit baking, as the result found a decrease in EGC and an increase in GCG in the baked shortbread biscuits. The effect of MGTP on shortbread biscuit acceptability was assessed using a 9-point hedonic scale and the data was analysed with response surface methodology. With an increase in the level of green tea powder, the acceptability of biscuits decreased significantly, and sugar did not significantly improve acceptability of the enriched biscuits. The main factors affecting acceptability were colour and bitterness. The consumption of MGTP as biscuit or tea infusion did not have significant effects on acute metabolic response, but some interesting trends were observed amongst a low number of Asian participants. This indicates potential for MGTP-enriched products as functional foods. An additional study with larger sample size that takes into account the ethnicity of participants is needed to confirm the observed trends.

## Figures and Tables

**Table 1:** Percentage retention of catechins and caffeine in MGTP (Matcha green tea powder)-enriched shortbread biscuits after baking as a proportion of initial content in the dough.

Compound	2 g MGTP (%)	4 g MGTP (%)	6 g MGTP (%)
(−)-epigallocatechin (EGC)	70.9 ± 9.5	81.5 ± 2.5	68.9 ± 1.6
(−)-epicatechin (EC)	72.6 ± 4.5	85.7 ± 3.5	80.2 ± 1.7
(−)-epigallocatechin gallate (EGCG)	84.9 ± 4.0	89.7 ± 2.1	81.8 ± 1.4
(+)-gallocatechin gallate (GCG)	133.0 ± 7.3	128.6 ± 11.0	140.3 ± 8.6
(−)-epicatechin gallate (ECG)	92.2 ± 3.9	94.3 ± 2.4	89.1 ± 1.2
Total catechins	82.4 ± 5.7	88.9 ± 2.5	81.1 ± 1.4
Caffeine	81.9 ± 5.0	76.3 ± 3.4	76.4 ± 1.1

## Supplementary Materials

The following are available online at http://www.mdpi.com/2304-8158/7/2/17/s1, Figure S1: Plain biscuit (**a**) and green tea biscuits with 3 levels of MGTP incorporated: (**b**) 2 g, (**c**) 4 g and (**d**) 6 g of MGTP 100 g^−1^ of flour, Figure S2: Chromatograph profiles of tea catechins, (**A**) in dough; (**B**) in plain and green tea biscuits: 1. EGC; 2. Caffeine; 3. EC; 4. EGCG; 5. GCG and 6. ECG, Figure S3: Hardness plot of biscuits with 3 levels of MGTP and sugar incorporated. The error bars represent the STDs (*n* = 6), Figure S4: Estimated nutritional data of food samples.
