# Supplementary material for: Enrichment of Biscuits with Matcha Green Tea Powder: Its Impact on Consumer Acceptability and Acute Metabolic Response
Source: Foods. 2018 Feb 1;7(2):17. doi: 10.3390/foods7020017 (PMC5848121; doi:10.3390/foods7020017)
Supplement: Supplementary file 1 [file foods-07-00017-s001.docx]

Food Samples

The nutritional information of food samples (Table 2) were calculated by using data from nutritional labels of ingredients and compensating with moisture loss after baking. Figure 1 shows the portions of biscuits in a plastic bag given to participants. Table 2 presents the amount of catechins and caffeine in MGTP used to produce green tea biscuits and drink for this study and the content of catechins and caffeine found in biscuits. The content of catechins and caffeine were determined by using HPLC method described earlier.

One portion of green tea biscuit samples (100 g) contained approximately 232.5 mg of total catechins, whereas one portion of green tea drink made with MGTP contained 256.8 mg of total catechins.

**Table S1.** Estimated nutritional data of food samples.

|  | Control | Green Tea Biscuits | Plain Biscuits with Green Tea Drink |
| --- | --- | --- | --- |
| Food samples | 100 g of plain biscuits with 300 mL of water | 100 g of green tea biscuits with 300 mL of water | 100 g of plain biscuits with green tea drink (used 3 g of MGTP dissolved in 200 mL of warm water ) and 100 mL of water |
| Carbohydrate (g) | 54.0 | 54.6 | 55.0 |
| Sugar (g) | 16.0 | 15.5 | 16.0 |
| Fat (g) | 36.0 | 35.15 | 36.15 |
| Total catechins (mg) | 0 | 232.5 | 256.8 |


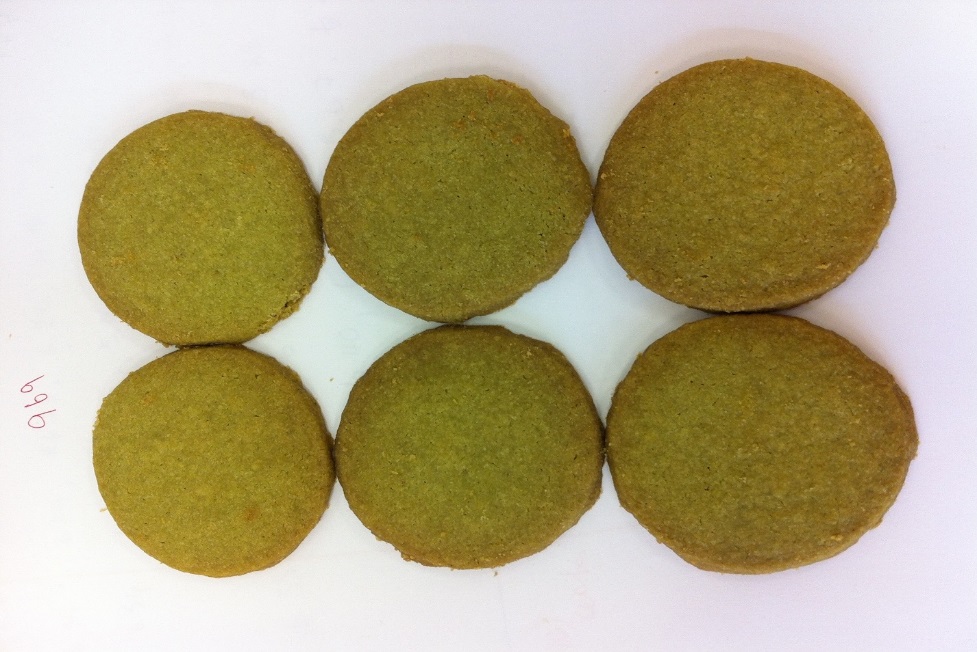

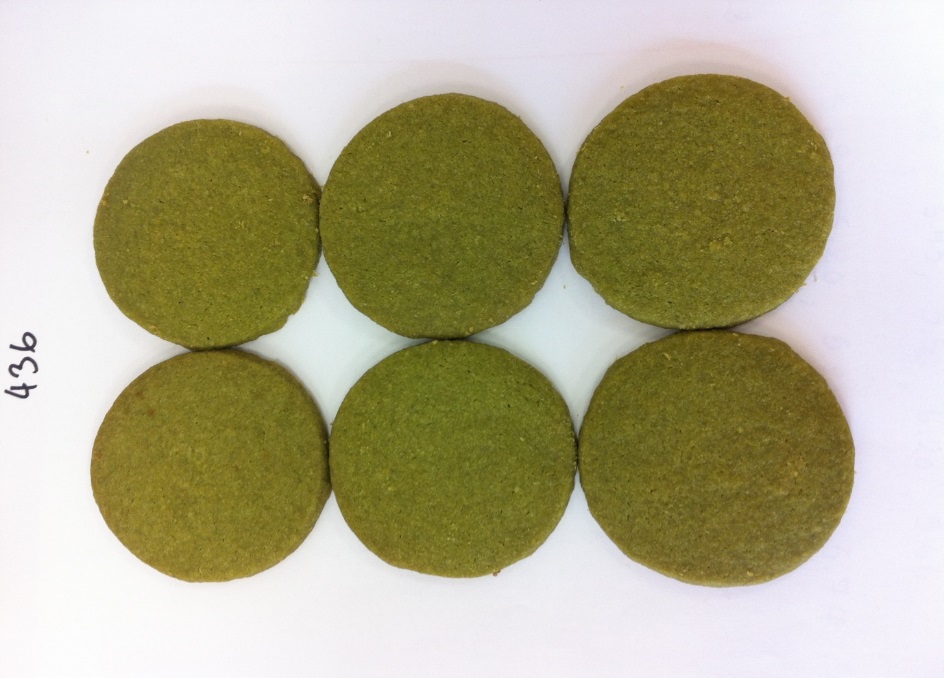

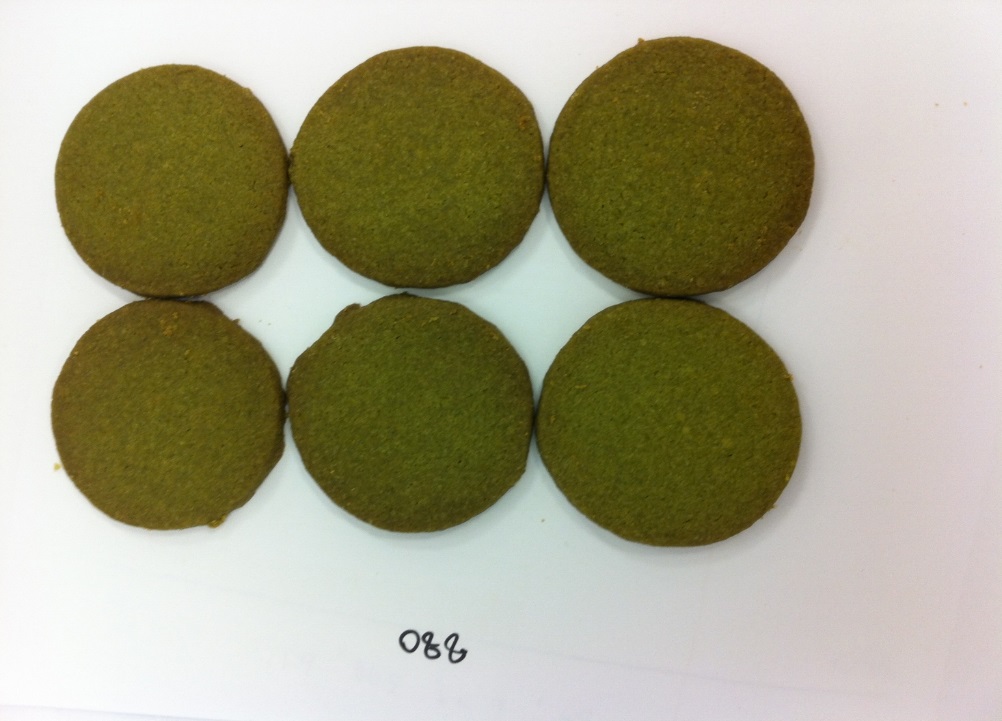

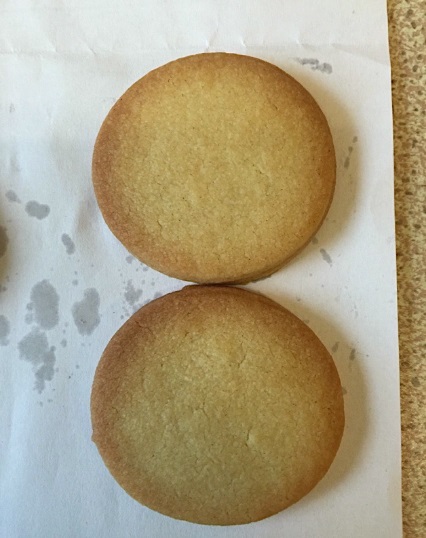


(a)

(b)

(c)

(d)

**Figure S1.** Plain biscuit (**a**) and green tea biscuits with 3 levels of MGTP incorporated, (**b**) 2 g, (**c**) 4 g and (**d**) 6 g of MGTP 100 g^−1^ of flour.

**Figure *S*2.** Chromatograph profiles of tea catechins, (**A**): in dough, (**B**): in plain and green tea biscuits: 1.EGC; 2.Caffeine; 3.EC; 4.EGCG; 5.GCG and 6.ECG.

**Figure S3.** Hardness plot of biscuits with 3 levels of MGTP and sugar incorporated. The error bars represent the STDs (*n* = 6)


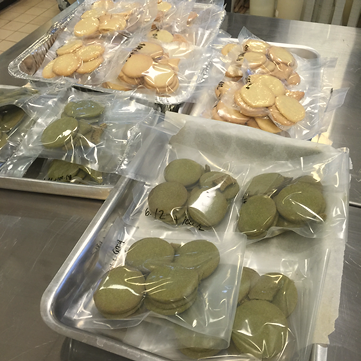


**Figure S4.** Packages of green tea biscuits and plain biscuits (100 g per pack) served to the subjects.
